# Real-world characteristics and invasive disease-free survival of resected patients with HR-positive/HER2-negative early breast cancer: evidence from German population-based cancer registries

**DOI:** 10.1016/j.esmorw.2026.100765

**Published:** 2026-09-02

**Authors:** P. Grieger, M.Y. Beykloo, C. Lee, J. Moreira, N. Niklas, D. Twardella, B. Köpf, D. Reil, C. Schneider, S. Luttmann, A. Nennecke, S.-Z. Kim-Wanner, F. Oesterling, B. Holleczek, R. Pritzkuleit, A. Katalinic, A. Waldmann

**Affiliations:** 1 Institute for Social Medicine and Epidemiology, University of Lübeck, Lübeck, Germany; 2 Data Science, Analytics and Technical Solutions, IQVIA, London, UK; 3 IQVIA, Oeiras, Portugal; 4 Oncology Evidence Network, IQVIA, Frankfurt, Germany; 5 Bavarian Cancer Registry, Munich, Germany; 6 Clinical-Epidemiological Cancer Registry Brandenburg-Berlin, Cottbus, Germany; 7 Bremen Cancer Registry, Bremen, Germany; 8 Hamburg Cancer Registry, Hamburg, Germany; 9 Hessian Cancer Registry, Hessian State Office for Health and Care, Frankfurt/Main, Germany; 10 Cancer Registry North Rhine-Westphalia, Bochum, Germany; 11 Saarland Cancer Registry, Saarbrücken, Germany; 12 Institute of Cancer Epidemiology, University of Lübeck, Lübeck, Germany

**Keywords:** early breast cancer, HER2-negative, HR-positive, invasive disease-free survival, real-world, recurrence

## Abstract

**Background:**

Hormone receptor-positive (HR-positive) and human epidermal growth factor receptor 2-negative (HER2-negative) tumors are the most prevalent subtypes of early breast cancer (eBC). Despite the generally favorable prognosis, a relevant proportion experiences invasive disease events following surgery. We aimed to describe characteristics, treatment modalities, and survival outcomes of surgically treated patients with HR-positive/HER2-negative eBC in Germany.

**Materials and methods:**

This study used data from eight population-based clinical cancer registries in Germany. Patients diagnosed between 2017 and 2021 with HR-positive/HER2-negative eBC who underwent surgery within 4 months of diagnosis were included. Patient and tumor characteristics and treatment received before recurrence were described. Kaplan–Meier statistics and multivariable Cox regression were used to assess invasive disease-free survival (iDFS).

**Results:**

A total of 43 214 patients with a median follow-up time of 47 months were included. Among these, 5635 experienced an iDFS event, of which 38.0% were recurrences as the first observed event. Recurrence more often occurred among men, premenopausal women, and patients with larger tumors, greater nodal involvement, and higher tumor grade. The 5-year iDFS was 82.9% (95% confidence interval 82.4-83.3). In multivariable analysis, higher age, tumor size, nodal involvement, histologic grade, and mastectomy compared with breast-conserving surgery were associated with a lower iDFS.

**Conclusions:**

These real-world data emphasize that 83% of patients with HR-positive/HER2-negative eBC do not experience recurrence, second primary malignancy, and death within 5 years. The prognostic relevance of established tumor-related factors for iDFS was confirmed in a large unselected patient population in the German routine care setting.

## Highlights

• Among patients with 43 214 HR-positive/HER2-negative eBC, 2142 experienced recurrence during follow-up.
• Recurrence was more frequent in male, premenopausal patients with advanced disease.
• The 3- and 5-year iDFS were 90.1% and 82.9%.
• iDFS varied across subgroups, with lower rates in higher-stage patients.
• Key prognostic factors for iDFS were confirmed in a German real-world cohort.

## Introduction

Breast cancer (BC) is the most common malignancy in Germany, accounting for 17.6% of all cancer-related deaths in women nationwide. In 2020, a total of 70 550 women and 740 men were diagnosed with BC, and 18 425 women and 166 men died due to BC.^1^

Among the molecular subtypes of BC, hormone receptor-positive (HR-positive)/human epidermal growth factor receptor 2-negative (HER2-negative) disease is the most common, accounting for ∼73% of all BC cases.^2^ The majority of all BCs are locoregional, with 90% of tumors corresponding to Union for International Cancer Control (UICC) stages I-III. Consequently, HR-positive/HER2-negative early breast cancer (eBC) is the most prevalent subtype.

The Current European Society for Medical Oncology (ESMO) guidelines recommend surgery as the initial treatment for patients with HR-positive/HER2-negative eBC, with the option of neoadjuvant therapy, followed by adjuvant endocrine therapy (ET) for a minimum duration of 5 years.^3^ The addition of chemotherapy (CT) and radiotherapy (RT) is guided by individual patient and tumor characteristics. Although ET has been shown to substantially reduce the risk of recurrence,^4^^,^^5^ a substantial residual risk remains. Evidence from a large meta-analysis of 88 randomized controlled trials indicates that patients with estrogen receptor-positive eBC, who complete 5 years of ET, still face a 15-year recurrence risk of 10%-41%, depending on tumor size, nodal involvement, and histologic grade.^6^ A German population-based study, including data from the cancer registry Saarland, reported 5- and 10-year recurrence rates of 8% and 14%, respectively, in patients with HR-positive BC.^7^

To capture metastatic or locoregional recurrence, second primary malignancy, and death from any cause, invasive disease-free survival (iDFS), as defined by the STEEP 2.0 Criteria,^8^ is increasingly used as a standardized surrogate endpoint for overall survival (OS). Compared with the less uniformly defined DFS, iDFS has emerged as the more relevant endpoint due to its standardized definition and widespread use in more recent trials.^9^ The risk of iDFS events can vary considerably according to patient- and tumor-related factors, such as tumor size, nodal involvement, menopausal status, and general health status. An analysis of US data from patients with stage II-III HR-positive/HER2-negative BC receiving adjuvant ET showed that the risk of an iDFS event was 26.1% at 5 years, increasing to 45.0% at 10 years.

Understanding the real-world burden of the iDFS events in the German setting is therefore of great clinical importance. However, large-scale real-world data remain limited, particularly from recent treatment periods. Although clinical trials provide a high level of evidence, they enroll patient populations which may not reflect the heterogeneity of patients treated in routine care. Population-based cancer registries offer a unique opportunity to overcome these limitations, providing comprehensive, unselected data on patient characteristics, treatment patterns, and clinical outcomes at a population level. Aggregating data across multiple registries further increases statistical power and geographic representativeness, enabling more robust estimates of real-world outcomes.

Using data from population-based clinical cancer registries in Germany, this study aims to describe the demographic and clinical characteristics of patients with stage I-III HR-positive/HER2-negative BC who underwent surgery within 4 months after diagnosis. We further aim to describe iDFS from the date of surgery and to identify associated risk factors.

## Materials and methods

This retrospective cohort study included patients with HR-positive/HER2-negative eBC identified from eight population-based cancer registries in Germany (Bavaria excluding the administrative district of Upper Bavaria and the city and the district of Landshut, Brandenburg-Berlin, Bremen, Hamburg, Hessen, North Rhine-Westphalia, Saarland, and Schleswig-Holstein), covering a total population of ∼45 million, equivalent to 54% of the German population. All registries operate under standardized federal and state-level legislation,^10^^,^^11^ ensuring uniform data collection on diagnoses, treatments, recurrences, and deaths. Treatment data are collected through mandatory reporting by all treating physicians and institutions. No linkage with external data sources was carried out. Data quality is ensured through internal validation procedures, including plausibility checks.

Patients were diagnosed between 1 January 2017 and 31 December 2021 and have been followed up until 31 December 2023, allowing for a minimum of 2 years of follow-up for all included patients. The diagnosis periods for each federal state are provided in Supplementary Table S1 (available at https://doi.org/10.1016/j.esmorw.2026.100765). Eligible patients were required to have an initial diagnosis of HR-positive/HER2-negative invasive BC [International Classification of Diseases Version 10 (ICD-10) code C50] within the study period and present with stage I-III disease at diagnosis, as determined from TNM (tumor–node–metastasis) variables according to the UICC eighth edition.^12^ Both male and female patients aged ≥18 years were included, provided they had undergone either breast-conserving surgery (BCS) or mastectomy or a combination of both within 4 months of initial diagnosis. In order to ensure a disease-free baseline for the assessment of iDFS, progression events were only considered if they were preceded by R0 resection. Recurrence events were included irrespective of residual status. This approach reflects changes in registry coding, as recurrence and progression were not distinguished within the overall assessment in earlier years.

Patients were also excluded if they had <6 months of follow-up, experienced metastatic recurrence within 3 months after initial diagnosis, or received HER2-targeted therapy (trastuzumab, trastuzumab emtansine, trastuzumab deruxtecan, pertuzumab, or lapatinib) between diagnosis and 3 months postsurgery. Additional exclusion criteria were any notification of recurrence, progression, or a second primary malignancy before surgery, any other primary malignancy (except nonmelanoma skin cancer, ICD-10 C44) within the 5 years preceding BC diagnosis, or missing vital status information. Patients with documented use of cyclin-dependent kinase (CDK) 4/6 inhibitors (palbociclib, ribociclib, or abemaciclib) before eBC diagnosis were also excluded, as such use would most likely reflect participation in a clinical trial rather than standard-of-care treatment. Data on CDK4/6 inhibitor exposure were not available for 7059 patients from Bavaria.

Recurrences during follow-up were derived from notification of both locoregional and metastatic recurrence. In addition, an algorithm was applied to detect further recurrences using information in notification on the occasion of surgery, RT, and systemic anticancer therapies (see Supplementary Methods S1, available at https://doi.org/10.1016/j.esmorw.2026.100765).

To analyze therapy received before recurrence, treatment records were used to identify CT, RT, and ET in relation to surgery. Neoadjuvant therapies included any treatments administered between the date of diagnosis and the first BCS or mastectomy. Adjuvant therapies were defined as treatments initiated from the date of surgery up to 274 days afterward. This extended window accounts for the delayed initiation of ET after the completion of CT and RT in real-world treatment sequences. Surgical procedures, RT, and ET were also analyzed, with ET classified as tamoxifen and/or aromatase inhibitor (AI).

Descriptive analyses were used to summarize baseline patient and tumor characteristics (sex, age, stage at diagnosis, tumor size, lymph node involvement, tumor grade, time from diagnosis to first surgery, menopausal, and local residual status after surgery), as well as to describe the treatment received before recurrence. The median follow-up time was estimated using the reverse Kaplan–Meier method.^13^

The primary outcome of the analysis was iDFS, which is defined as the period from the date of the first surgery until the earliest occurrence of metastatic or locoregional recurrence, second primary malignancy of any type [excluding nonmelanoma skin cancer (ICD-10 C44)], or death from any cause. OS was defined as the time from the date of first surgery to the date of death, in order to ensure comparability between the endpoints. Data were analyzed using the Kaplan–Meier method. The number of patients remaining at risk and the corresponding probabilities of not having experienced an event were reported up to 6 years after the first surgery. The survival analyses were further stratified by menopausal status, tumor size, nodal involvement, and tumor stage in exploratory subgroup analyses.

Cox proportional-hazards regression models were used to evaluate the impact of patient and tumor characteristics and treatment-related variables on iDFS. Adjusted hazard ratios (HRs) were reported with corresponding 95% confidence intervals (CIs) and *P* values derived from Wald tests. Patients were censored at the date of last follow-up if no iDFS event had occurred. The proportional hazards assumption was assessed graphically using Schoenfeld residuals (Supplementary Figure S1, available at https://doi.org/10.1016/j.esmorw.2026.100765). Variables for the multivariable Cox regression model were selected a priori based on their clinical relevance and known prognostic importance in eBC while avoiding the simultaneous inclusion of highly correlated variables. Potential multicollinearity was assessed using the variance inflation factor. Treatment variables with suspected substantial underreporting in registry data, including adjuvant ET, RT, and CT, were excluded from the analysis. Missing values were retained in the analyses and included as a separate category (‘missing’) for the respective covariate. Univariable Cox proportional hazards models were fitted to estimate unadjusted associations between each covariate and iDFS.

To evaluate the robustness of the primary multivariable model with respect to coefficient stability and potential overfitting arising from correlated covariates, a sensitivity analysis using penalized Cox regression with the least absolute shrinkage and selection operator was carried out.

All statistical analyses were run with R 4.1.3^14^ using the packages *survival*,^15^ *survminer*,^16^ *finalfit*,^17^ and *forestplot*.^18^

The study complied with the Declaration of Helsinki and was reported to the ethics committee of the University of Lübeck (Az 2024-206). The study is registered in the German Clinical Trials Register (DRKS00033796). European Society of Medical Guidance for Reporting Oncology real-world Evidence guidelines were followed.^19^

## Results

### Patient demographic and clinical information

**Figure 1:**
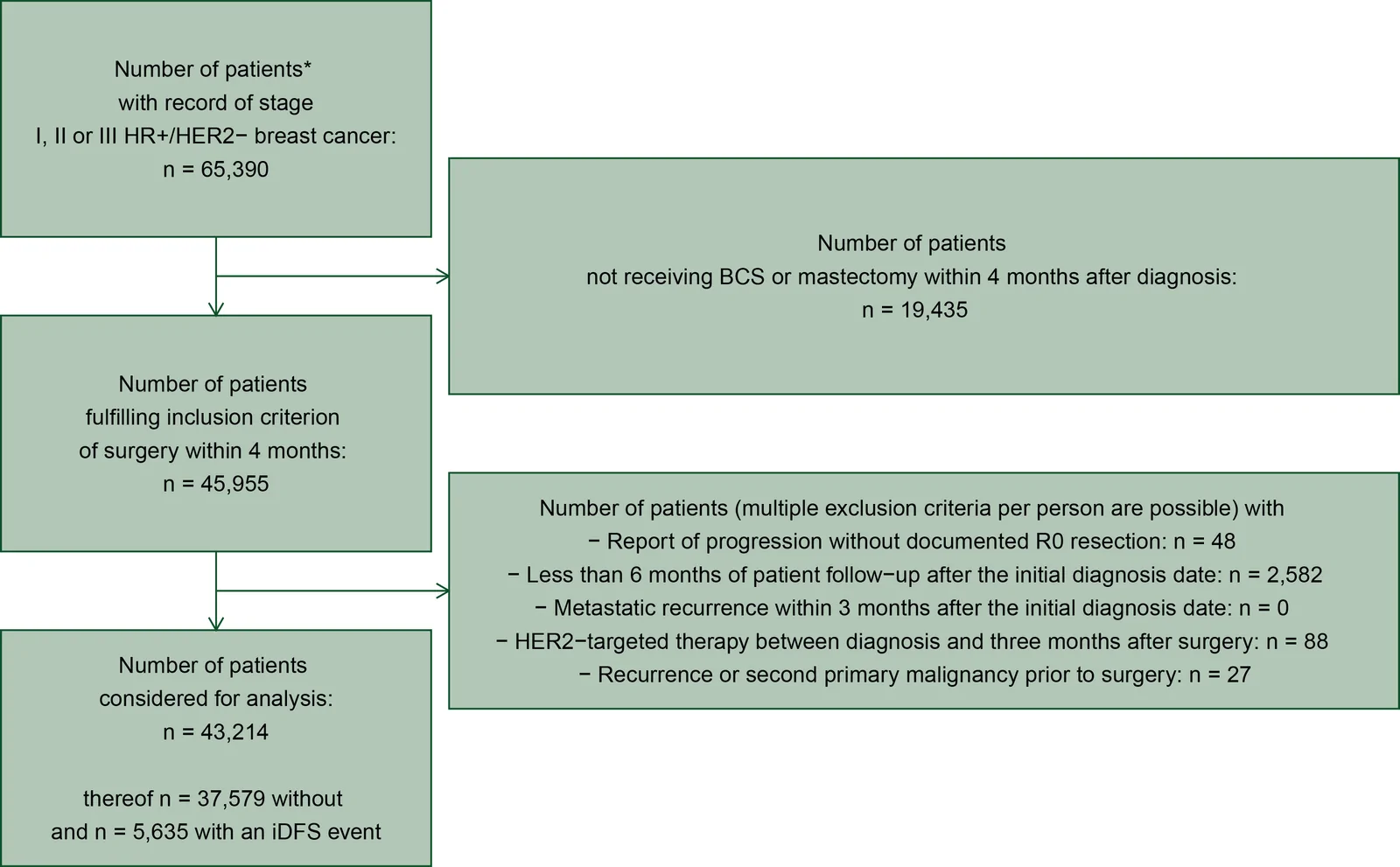
**Flowchart with inclusion and exclusion criteria.** ∗Patients were required to have no other primary malignancy (except nonmelanoma skin cancer, ICD-10 C44) within 5 years preceding breast cancer diagnosis, no notification of recurrence or a second primary malignancy before surgery, no missing vital status information at the end of follow-up, and no prior receipt of cyclin dependent kinase 4/6 inhibitors (palbociclib, ribociclib, or abemaciclib; except for Bavaria). As these criteria had already been applied by some registries before data delivery, it was not possible to report the exact number of exclusions for each criterion separately in the flow chart. BCS, breast-conserving surgery; HR, hormone receptor; HER, human epidermal growth factor receptor; ICD, International Classification of Diseases.

A total of 65 390 patients diagnosed with HR-positive/HER2-negative eBC between 2017 and 2021 were identified from the cancer registries. Following the application of the inclusion and exclusion criteria, a total of 43 214 patients with HR-positive/HER2-negative eBC were included in the analysis (Figure 1). The median age at diagnosis was 64 years and 0.9% of the study population was male. During the follow-up period, 5635 patients experienced an iDFS event, 38.0% of which were recurrences. Patients who experienced an iDFS event were more frequently diagnosed with advanced-stage disease and higher tumor grade compared with those without an iDFS event. Among patients with an iDFS event, the subgroup with recurrence showed an even higher proportion of stage III disease (28.3% among patients with recurrence versus 7.7% without an iDFS event), tumor grade 3 (26.5% versus 12.3%), and R1 resection status after first surgery (16.7% versus 11.1%) (Table 1).

**Table 1:** Demographic and clinical characteristics overall and by iDFS status

Characteristic	Total (*N* = 43 214)	No iDFS events (*n* = 37 579)	iDFS event	
			All iDFS events (*n* = 5635)	Recurrence (*n* = 2142)
Sex, *n* (%)				
Female	42 830 (99.1)	37 301 (99.3)	5529 (98.1)	2110 (98.5)
Male	384 (0.9)	278 (0.7)	106 (1.9)	32 (1.5)
Age at diagnosis, median (IQR)	64 (54-74)	63 (54-73)	72 (60-80)	64 (52-76)
Age categories (years), *n* (%)				
18-44	2460 (5.7)	2159 (5.7)	301 (5.3)	246 (11.5)
45-64	19 680 (45.5)	18 103 (48.2)	1577 (28.0)	855 (39.9)
65-74	10 563 (24.4)	9292 (24.7)	1271 (22.6)	443 (20.7)
75-79	5233 (12.1)	4283 (11.4)	950 (16.9)	276 (12.9)
80-84	3853 (8.9)	2889 (7.7)	964 (17.1)	237 (11.1)
≥85	1425 (3.3)	853 (2.3)	572 (10.2)	85 (4.0)
Stage at diagnosis, *n* (%)				
Stage I	21 786 (50.4)	20 060 (53.4)	1726 (30.6)	493 (23.0)
Stage IIA	11 752 (27.2)	10 120 (26.9)	1632 (29.0)	605 (28.2)
Stage IIB	5513 (12.8)	4505 (12.0)	1008 (17.9)	429 (20.0)
Stage III	4163 (9.6)	2894 (7.7)	1269 (22.5)	615 (28.7)
Tumor size at diagnosis (T), *n* (%)				
T0	11 (0.0)	10 (0.0)	1 (0.0)	0 (0)
T1	25 123 (58.1)	23 002 (61.2)	2121 (37.6)	685 (32.0)
T2	14 873 (34.4)	12 285 (32.7)	2588 (45.9)	1066 (49.8)
T3	2393 (5.5)	1782 (4.7)	611 (10.8)	292 (13.6)
T4	814 (1.9)	500 (1.3)	314 (5.6)	99 (4.6)
Lymph node involvement at diagnosis (*N*), *n* (%)				
N0	31 058 (71.9)	27 833 (74.1)	3225 (57.2)	1008 (47.1)
N1	9397 (21.7)	7873 (21.0)	1524 (27.0)	663 (31.0)
N2+	2759 (6.4)	1873 (5.0)	886 (15.7)	471 (22.0)
Tumor grade at diagnosis, *n* (%)				
G1	8722 (20.2)	7994 (21.3)	728 (12.9)	190 (8.9)
G2	28 314 (65.5)	24 688 (65.7)	3626 (64.3)	1370 (64.0)
G3	5842 (13.5)	4606 (12.3)	1236 (21.9)	567 (26.5)
Missing	336 (0.8)	291 (0.8)	45 (0.8)	15 (0.7)
Menopausal status at diagnosis^a^, *n* (%)				
Premenopausal	10 570 (24.7)	9639 (25.8)	931 (16.8)	629 (29.8)
Postmenopausal	32 260 (75.3)	27 662 (74.2)	4598 (83.2)	1481 (70.2)
Time from diagnosis to first surgery (months), median (IQR)	1.1 (0.8-1.6)	1.1 (0.8-1.6)	1.1 (0.7-1.6)	1.1 (0.7-1.6)
Local residual status after first surgery, *n* (%)				
R0	37 523 (86.8)	32 698 (87.0)	4825 (85.6)	1745 (81.5)
R1	4861 (11.2)	4155 (11.1)	706 (12.5)	357 (16.7)
R2	16 (0.0)	12 (0.0)	4 (0.1)	0 (0)
Missing	814 (1.9)	714 (1.9)	100 (1.8)	40 (1.9)

iDFS, invasive disease-free survival; IQR, interquartile range.

a Menopausal status analyzed for female patients only.

### Treatment modalities

Documented treatment modalities are summarized in Table 2. Although the use of neoadjuvant therapies was comparable between patients with and without an iDFS event, disparities were evident in the surgical approach, adjuvant CT, and RT treatment. Mastectomy was carried out more frequently among patients who experienced an iDFS event (36.8% versus 17.9%). The proportion of patients undergoing a combined surgical approach involving both BCS and mastectomy was highest in the recurrence subgroup (15.3%). Adjuvant CT was observed more frequently among patients with an iDFS event (12.6% versus 3.8%), with the highest rate among patients who experienced a recurrence (28.6%). Furthermore, 41.3% of patients received adjuvant ET, predominantly AIs, which were prescribed to postmenopausal women in 89.7% of cases.

**Table 2:** Documented treatment received before recurrence overall and by iDFS status

Treatment	Total (*n* = 43 214)	No iDFS events (*n* = 37 579)	iDFS events	
			All iDFS events (*n* = 5635)	Recurrence (*n* = 2142)
Neoadjuvant CT, *n* (%)	77 (0.2)	55 (0.1)	22 (0.4)	9 (0.4)
Neoadjuvant ET, *n* (%)	1285 (3.0)	1121 (3.0)	164 (2.9)	72 (3.4)
Type of surgery, *n* (%)				
BCS	32 783 (75.9)	29 651 (78.9)	3132 (55.6)	1115 (52.1)
Mastectomy	8802 (20.4)	6731 (17.9)	2071 (36.8)	700 (32.7)
BCS and mastectomy	1629 (3.8)	1197 (3.2)	432 (7.7)	327 (15.3)
Adjuvant CT^a^, *n* (%)	2126 (4.9)	1416 (3.8)	710 (12.6)	612 (28.6)
Any RT, *n* (%)	28 084 (65.0)	25 019 (66.6)	3065 (54.4)	1301 (60.7)
Adjuvant ET only^b^, *n* (%)	3757 (8.7)	3108 (8.3)	649 (11.5)	182 (8.5)
Type of ET received, *n* (%)				
AI	8600 (19.9)	7315 (19.5)	1285 (22.8)	520 (24.3)
Tamoxifen	7173 (16.6)	6453 (17.2)	720 (12.8)	307 (14.3)
Tamoxifen and AI	2071 (4.8)	1802 (4.8)	269 (4.8)	134 (6.3)
None or not documented	25 370 (58.7)	22 009 (58.6)	3361 (59.6)	1181 (55.1)

AI, aromatase inhibitor; BCS, breast-conserving surgery; CT, chemotherapy; ET, endocrine therapy; iDFS, invasive disease-free survival; RT, radiotherapy.

a Based on a record of CT between surgery date and 274 days afterward.

b Based on a record of ET with no record of CT or RT after the date of disease diagnosis.

### Oncological outcomes

During the follow-up period (median, 47 months; interquartile range, 35-61), 3156 patients died, corresponding to a 3-year OS of 95.0% (95% CI 94.8-95.3) and a 5-year OS of 89.9% (95% CI 89.5-90.2). OS curves including stratified analyses by menopausal status, tumor size, nodal involvement, and tumor stage are shown in Supplementary Figure S2 (available at https://doi.org/10.1016/j.esmorw.2026.100765).

**Figure 2:**
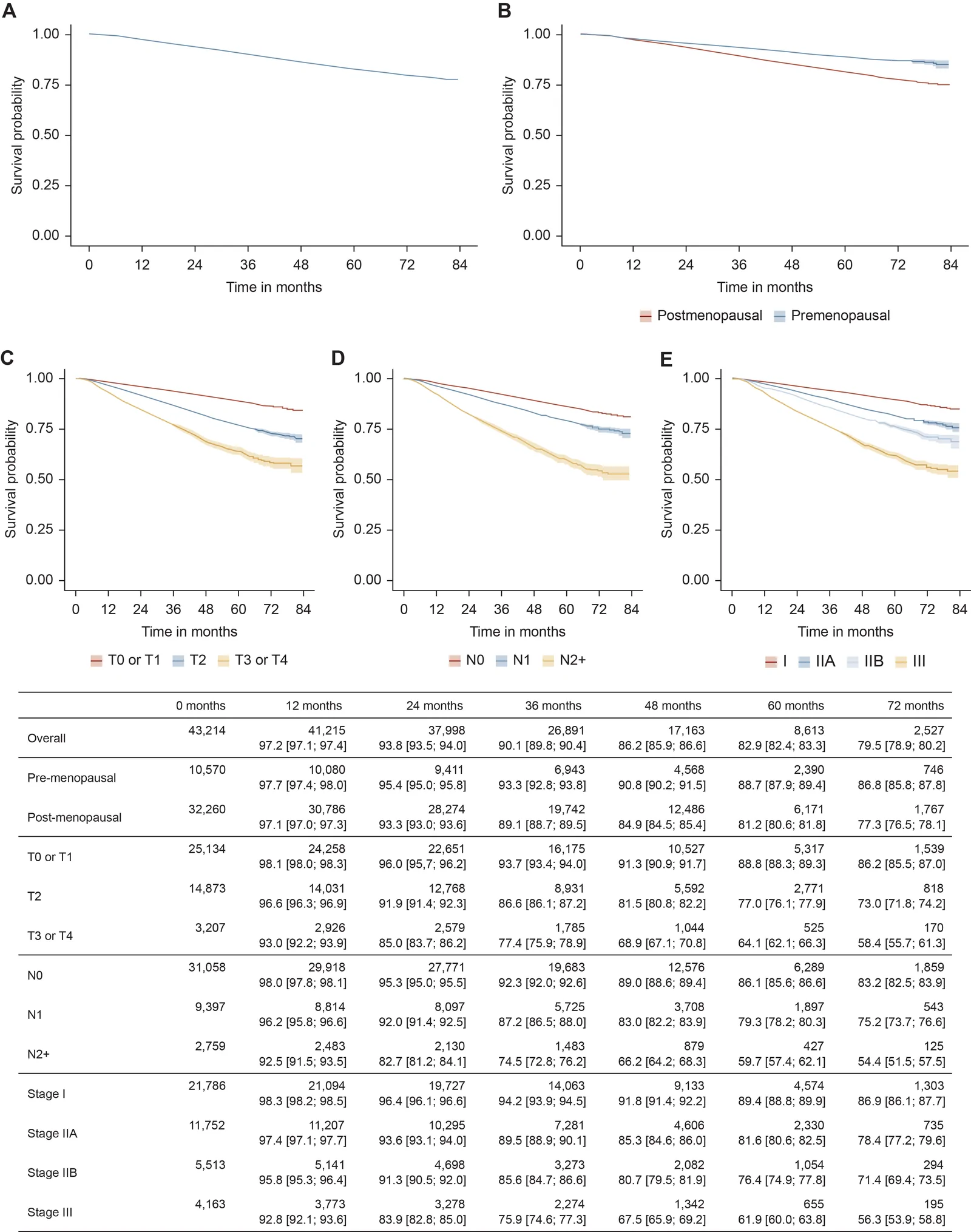
**Invasive disease-free survival probabilities overall (A) and by menopausal status (B), tumor size (C), nodal involvement (D), and Union for International Cancer Control stage (E) with corresponding numbers at risk and survival probabilities (%), including 95% confidence intervals at selected time points**.

**Figure 3:**
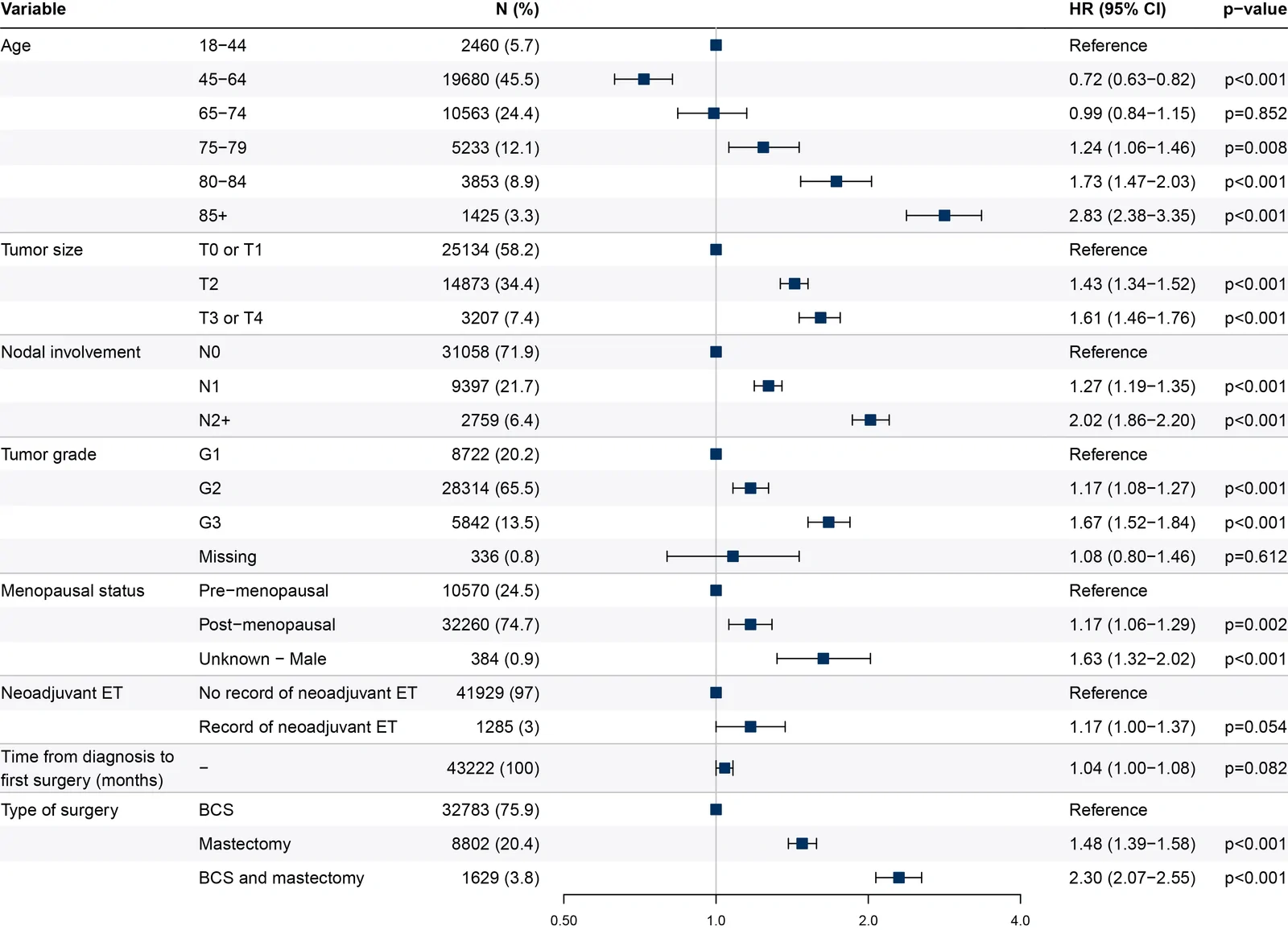
**Forest plot of the multivariable Cox proportional hazards regression model for invasive disease-free survival, showing the number of patients (*N*), HRs with 95% CIs, and corresponding *P* values**. BCS, breast-conserving surgery; CI, confidence interval; ET, endocrine therapy; HR, hazard ratio.

Of 5635 patients who experienced an iDFS event, the first observed event was recurrence in 2142 patients (38.0%), a second primary malignancy in 1415 patients (25.1%), and 2078 patients (36.9%) died without evidence of recurrence or a second primary malignancy in the cancer registry dataset. In the HR-positive/HER2-negative eBC cohort, the iDFS was 90.1% at 3 years and 82.9% at 5 years (Figure 2). Premenopausal women had a higher 5-year iDFS compared with postmenopausal women (88.7% versus 81.2%). This finding was confirmed in multivariable Cox regression (Figure 3), with postmenopausal status associated with a higher risk of an iDFS event compared with premenopausal status (HR 1.19, 95% CI 1.08-1.31) after adjustment for tumor- and patient-related characteristics. Five-year iDFS declined with advancing tumor stage and with increasing tumor size and nodal involvement. This association remained significant in multivariable Cox regression, in which larger tumor size and higher nodal involvement were independently associated with an increased hazard of an iDFS event.

Age categories were associated with iDFS in multivariable Cox analysis. Patients aged 45-64 years experienced a lower hazard of iDFS events compared with the youngest age group (HR 0.76, 95% CI 0.66-0.86), whereas the hazard increased significantly in patients aged ≥75 years. Type of surgery was also significantly associated with iDFS in multivariable analyses. Mastectomy (HR 1.40, 95% CI 1.31-1.50) and the combined surgical approach involving BCS followed by mastectomy (HR 2.08, 95% CI 1.87-2.32) were associated with higher hazards compared with BCS.

Additional variables not included in the multivariable model, such as residual status and UICC stage, also showed associations with iDFS in the univariable analysis (Supplementary Figure S3, available at https://doi.org/10.1016/j.esmorw.2026.100765). Sensitivity analyses using penalized Cox regression confirmed the stability of the adjusted model, with nonzero coefficients retained for all covariates.

## Discussion

We requested, harmonized,^20^ and pooled data from eight population-based cancer registries in Germany in order to describe the real-world characteristics and clinical outcomes of patients with HR-positive/HER2-negative eBC. This subtype is the most prevalent among eBC and is associated with the highest iDFS and OS in the initial years following diagnosis.^21^ Despite the generally favorable prognosis of this subtype, several studies have described that a relevant proportion of patients still experience disease recurrence.^6^^,^^7^^,^^22^

Based on our large, population-based sample of 43 214 patients with HR-positive/HER2-negative eBC who underwent surgery, 5635 patients (13.1%) experienced an iDFS event during the follow-up period of the study. Among them were 2142 patients (5.0%) who experienced a recurrence as the first observed event. Patients with an iDFS event differed from those without an event in both patient- and tumor-related characteristics, including higher age, likely reflecting the inclusion of death in the composite endpoint. Among patients with recurrence, a higher proportion of men and premenopausal women as well as more advanced UICC stage and higher tumor grade were observed compared with patients without an iDFS event. Consistent with these descriptive findings, the multivariable Cox proportional hazards model identified increasing age (among patients aged ≥75 years), larger tumor size, nodal involvement, and higher tumor grade as predictors of worse iDFS. These results are in line with previous analyses in patients with HR-positive/HER2-negative eBC, which consistently identified tumor size and lymph node involvement as predictors for adverse outcomes, including recurrence.^23^^,^^24^ Although an association between increasing age and recurrence was not observed in all studies,^23^ other real-world cohorts reported a higher recurrence risk among younger women. In particular, Walbaum et al.^25^ described that younger age is associated with higher recurrence risk scores, supporting our observation of an increased hazard among younger patients compared with those aged 45-64 years.

Among the treatment-related variables, the results of the multivariable Cox model mostly confirmed the patterns observed in the descriptive analyses. A higher proportion of patients who experienced iDFS events underwent mastectomy or a combined surgical approach. Accordingly, these variables were associated with an increased hazard of iDFS events in the multivariable analysis. However, their interpretation must take potential residual confounding into account. Patients undergoing mastectomy typically present with higher-risk disease characteristics that are not fully captured by the variables included in the cancer registry, and thus, in the model.^26^ In particular, important prognostic and predictive factors such as Ki-67, genomic risk scores, comorbidities, and other molecular tumor features were not systematically documented within the nationwide standardized dataset^10^ and could not be included in the analysis. Even after multivariable adjustment, residual confounding may therefore persist.

The administration of neoadjuvant therapy to 3.0% of our patients is consistent with the findings of a previous Finnish real-world study, which reported a rate of 2.7% in a comparable cohort.^21^ Adjuvant ET was administered to 41.3% of patients, which is markedly lower than the 79%-81% reported in comparable real-world studies,^21^^,^^27^ most likely reflecting underreporting of treatment applied in the outpatient setting in German registry data. Reports from the audit of certified BC centers in Germany document that ∼85% of all certified BC centers meet the target that ≥95% of all patients with estrogen receptor-positive BC are recommended ET.^28^ Similarly, the observed rates of adjuvant CT and RT in our cohort are likely to underestimate true treatment uptake, possibly reflecting incomplete reporting of these treatments in clinical cancer registry data.

iDFS were analyzed to evaluate long-term prognosis of patients with HR-positive/HER2-negative eBC and to capture recurrence, second primary malignancy, and death. Direct comparison of reported iDFS rates in the international literature is difficult due to differences in inclusion criteria and the stratification into low-, intermediate-, and high-risk groups based on tumor size, nodal involvement, and tumor grade. Published 5-year iDFS rates of European studies range from 73% to 89%,^29^^,^^30^ which is broadly consistent with our findings (82.9%). O’Shaughnessy et al.^22^ reported a 5-year risk of iDFS events of 22.7% for stage II and 40.4% for stage III patients, which is comparable to the 5-year iDFS of 81.6% for stage IIA, 76.4% for stage IIB, and 61.9% for stage III patients in our cohort. Another US study reported a 2-year iDFS of 97.1% and 5-year iDFS of 92.0% for node-negative patients,^31^ which is higher than the corresponding rates observed in our cohort (95.3% and 86.1%). However, this study did not include second primary malignancies in its endpoint.

### Strengths and limitations

In Germany, clinical cancer registration is legally mandated and conducted at the federal state level, with nationwide standardized data elements collected.^10^^,^^32^ Treating physicians are obliged to report relevant clinical events, including disease progression, recurrence, and death. In addition, registry data are regularly linked with population registry offices and systematically validated against death certificates within each federal state. The involvement of multiple registries from federal states of different sizes and geographic regions enhances the representativeness and generalizability of the findings. Moreover, the absence of restrictive inclusion and exclusion criteria, as typically applied in randomized controlled trials, supports the external validity of our results and their applicability to routine clinical practice.

There are some limitations to this study. The analysis was retrospective and descriptive, based on data from multiple population-based cancer registries with different inclusion periods and follow-up durations. In addition, parts of the follow-up period overlapped with the coronavirus disease pandemic, which may have influenced treatment decisions and/or disrupted planned therapies. Given the median follow-up time of 47 months in this cohort, a substantial number of late recurrences may not have been captured. Population-based analyses have shown that distant recurrences of estrogen receptor-positive BC continue to occur at a consistent rate for >5 years after diagnosis.^6^ These observations highlight the importance of reevaluating the findings once more extensive registry-based follow-up data are available in Germany.

The restriction of the study to patients who underwent surgery within 4 months of diagnosis may introduce a degree of selection bias, as patients with a longer time to surgery (8% of operated patients) were excluded from the study. These patients may differ systematically from the included population, for example by having received longer neoadjuvant therapy or by presenting with more advanced disease, which could potentially lead to an overestimation of iDFS in our cohort.

The use of real-world data carries an inherent risk of missing or incomplete treatment information. In our cohort, the proportion of patients receiving adjuvant ET was comparatively low, which may most likely reflect underreporting of therapies delivered in the outpatient setting, in which endocrine treatment is predominantly prescribed and monitored. Similar limitations apply to the reporting of recurrence events, which is also described for Danish Cancer Registry data.^29^ To overcome this latter limitation, a predefined algorithm based on available treatment information was used to identify recurrence events in addition to the reported events (Supplementary Methods S1, available at https://doi.org/10.1016/j.esmorw.2026.100765). The algorithm identified additional recurrence events, increasing the proportion of patients experiencing an iDFS event from 12.1% to 13.0%. The difference in the 5-year iDFS rate before and after the application of the algorithm (83.9% versus 82.9%) suggests that its impact on iDFS estimates was limited. However, the algorithm’s performance depends on the completeness of documented treatment information and it has not been externally validated, leaving the possibility of misclassifying recurrence events. Furthermore, according to registry coding practices, invasive recurrences following a prior in situ tumor are recorded as new tumor cases and are therefore not captured as recurrences.

These considerations underline the critical role of complete and precise treatment documentation in population-based cancer registries. Improving the capture of therapy information would enhance the validity of real-world analyses and strengthen their value for clinical decision-making.

### Conclusion

This population-based study provides comprehensive real-world evidence on patient characteristics, treatment patterns, and clinical outcomes among patients with HR-positive/HER2-negative eBC in Germany. Using data from multiple population-based federal state cancer registries, our study offers insight into routine clinical practice beyond the setting of randomized controlled trials. Despite the generally favorable prognosis of HR-positive/HER2-negative eBC, a relevant risk of recurrence remains. Even among node-negative patients, the 5-year iDFS remained below 90%, highlighting opportunities to further improve outcomes in this broad patient population.

Overall, these real-world data highlight the importance of ongoing evaluation of risk-adapted treatment strategies to improve outcomes for patients with HR-positive/HER2-negative eBC.

## Declaration of AI and AI-Assisted technologies in the writing process

During the preparation of this work, the authors used ChatGPT (OpenAI, San Francisco, CA, USA) for language editing and text refinement. After using this tool, the authors reviewed and edited the content as needed and take full responsibility for the content of the publication.
