# Supplementary material for: Real-world characteristics and invasive disease-free survival of resected patients with HR-positive/HER2-negative early breast cancer: evidence from German population-based cancer registries
Source: ESMO Real World Data Digit Oncol. 2026 Sep 2;13:100765. doi: 10.1016/j.esmorw.2026.100765 (PMC13571318; doi:10.1016/j.esmorw.2026.100765)
Supplement: Supplementary file 1 [file mmc1.pdf]

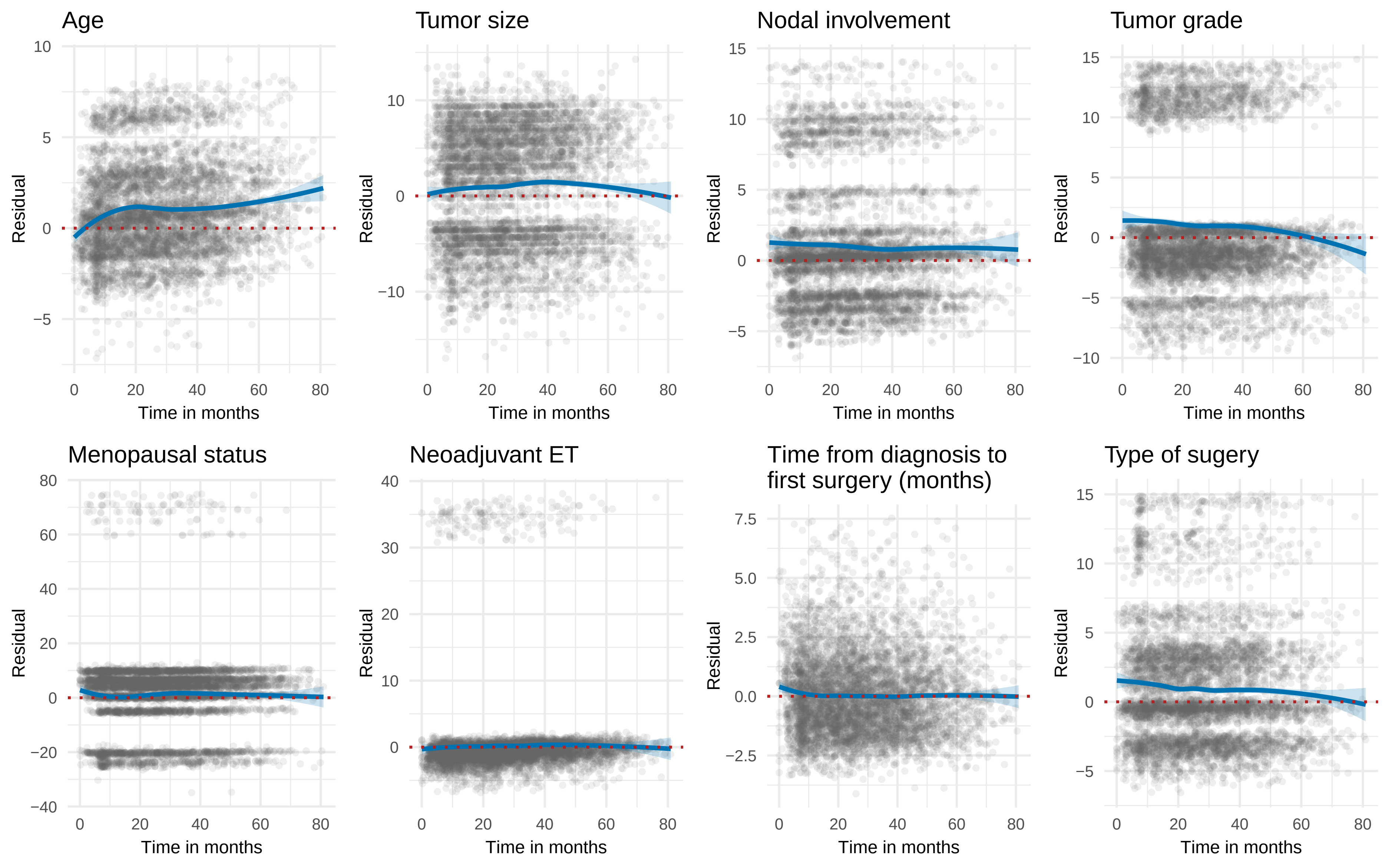

Age
Tumor size
Nodal involvement
Tumor grade
Menopausal status
Neoadjuvant ET
Time from diagnosis to first surgery (months)
Type of sugery
Residual
Time in months
