# Supplementary material for: Real-world characteristics and invasive disease-free survival of resected patients with HR-positive/HER2-negative early breast cancer: evidence from German population-based cancer registries
Source: ESMO Real World Data Digit Oncol. 2026 Sep 2;13:100765. doi: 10.1016/j.esmorw.2026.100765 (PMC13571318; doi:10.1016/j.esmorw.2026.100765)
Supplement: Supplementary file 2 [file mmc2.pdf]

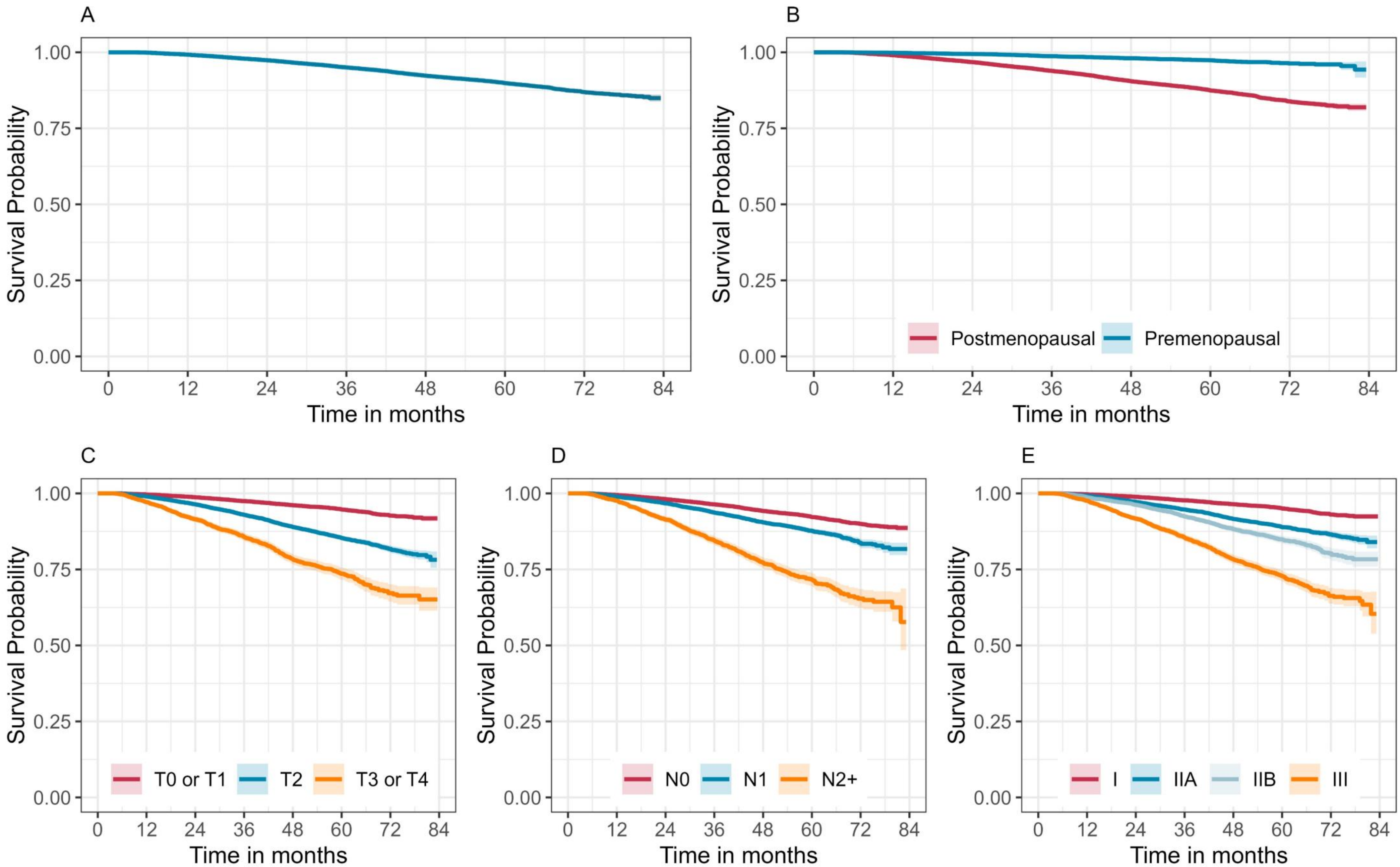

	0 months	12 months	24 months	36 months	48 months	60 months	72 months
Overall	43,214	42,033<br>99.2 [99.1; 99.3]	39,414<br>97.4 [97.2; 97.5]	28,318<br>95.0 [94.8; 95.3]	18,368<br>92.3 [92.0; 92.6]	9,358<br>89.9 [89.5; 90.2]	2,790<br>86.9 [86.4; 87.5]
Pre-menopausal	10,570	10,297<br>99.8 [99.8; 99.9]	9,793<br>99.4 [99.3; 99.6]	7,334<br>98.7 [98.5; 99.0]	4,922<br>98.0 [97.7; 98.4]	2,626<br>97.4 [97.0; 97.8]	838<br>96.3 [95.7; 97.0]
Post-menopausal	32,260	31,374<br>99.1 [99.0; 99.2]	29,288<br>96.8 [96.6; 97.0]	20,759<br>93.9 [93.6; 94.2]	13,311<br>90.5 [90.1; 90.9]	6,663<br>87.5 [87.0; 87.9]	1,932<br>83.9 [83.1; 84.6]
T0 or T1	25,134	24,614<br>99.6 [99.6; 99.7]	23,271<br>98.7 [98.6; 98.9]	16,781<br>97.5 [97.3; 97.7]	11,051<br>96.1 [95.8; 96.4]	5,666<br>94.7 [94.3; 95.1]	1,668<br>92.7 [92.1; 93.3]
T2	14,873	14,367<br>99.0 [98.8; 99.2]	13,369<br>96.4 [96.1; 96.7]	9,567<br>92.9 [92.5; 93.4]	6,137<br>88.9 [88.3; 89.5]	3,100<br>85.3 [84.5; 86.1]	930<br>81.7 [80.6; 82.9]
T3 or T4	3,207	3,052<br>97.2 [96.6; 97.7]	2,774<br>91.5 [90.5; 92.5]	1,970<br>85.7 [84.4; 86.9]	1,180<br>78.3 [76.7; 80.0]	592<br>73.6 [71.6; 75.6]	192<br>67.2 [64.4; 70.1]
N0	31,058	30,347<br>99.5 [99.4; 99.5]	28,564<br>98.1 [97.9; 98.3]	20,505<br>96.4 [96.2; 96.6]	13,304<br>94.2 [93.9; 94.5]	6,737<br>92.2 [91.8; 92.6]	2,021<br>90.0 [89.3; 90.6]
N1	9,397	9,070<br>99.0 [98.8; 99.2]	8,497<br>96.7 [96.4; 97.1]	6,142<br>93.7 [93.1; 94.2]	4,046<br>90.4 [89.7; 91.1]	2,110<br>87.7 [86.8; 88.6]	618<br>83.7 [82.3; 85.1]
N2+	2,759	2,616<br>97.6 [97.0; 98.2]	2,353<br>91.4 [90.4; 92.5]	1,671<br>84.4 [83.0; 85.9]	1,018<br>77.2 [75.4; 79.1]	511<br>71.6 [69.4; 73.8]	151<br>65.4 [62.4; 68.5]
Stage I	21,786	21,364<br>99.7 [99.6; 99.7]	20,216<br>98.8 [98.7; 99.0]	14,547<br>97.7 [97.5; 97.9]	9,566<br>96.5 [96.2; 96.7]	4,859<br>95.0 [94.6; 95.4]	1,407<br>93.0 [92.4; 93.7]
Stage IIA	11,752	11,413<br>99.2 [99.1; 99.4]	10,677<br>97.2 [96.9; 97.5]	7,696<br>94.7 [94.3; 95.1]	4,962<br>91.6 [91.0; 92.2]	2,544<br>88.9 [88.1; 89.7]	810<br>86.5 [85.5; 87.6]
Stage IIB	5,513	5,295<br>98.7 [98.5; 99.0]	4,943<br>96.3 [95.7; 96.8]	3,531<br>92.4 [91.7; 93.2]	2,291<br>88.2 [87.2; 89.2]	1,189<br>84.9 [83.6; 86.1]	342<br>80.3 [78.4; 82.2]
Stage III	4,163	3,961<br>97.6 [97.1; 98.1]	3,578<br>91.7 [90.8; 92.5]	2,544<br>85.4 [84.3; 86.6]	1,549<br>78.3 [76.9; 79.8]	766<br>72.8 [71.0; 74.6]	231<br>66.3 [63.8; 68.9]
