# Supplementary material for: Real-world characteristics and invasive disease-free survival of resected patients with HR-positive/HER2-negative early breast cancer: evidence from German population-based cancer registries
Source: ESMO Real World Data Digit Oncol. 2026 Sep 2;13:100765. doi: 10.1016/j.esmorw.2026.100765 (PMC13571318; doi:10.1016/j.esmorw.2026.100765)

Variable		N (%)	HR (95% CI)	p−value
Sex	Female	42830 (99.1)	Reference	
	Male	384 (0.9)	2.45 (2.02−2.97)	p<0.001
Age	18−44	2460 (5.7)	Reference	
	45−64	19680 (45.5)	0.65 (0.57−0.73)	p<0.001
	65−74	10563 (24.4)	0.98 (0.87−1.11)	p=0.789
	75−79	5233 (12.1)	1.46 (1.28−1.66)	p<0.001
	80−84	3853 (8.9)	2.23 (1.96−2.54)	p<0.001
	85+	1425 (3.3)	3.99 (3.47−4.59)	p<0.001
Tumor size	T0 or T1	25134 (58.2)	Reference	
	T2	14873 (34.4)	2.18 (2.06−2.31)	p<0.001
	T3 or T4	3207 (7.4)	3.86 (3.58−4.17)	p<0.001
Nodal involvement	N0	31058 (71.9)	Reference	
	N1	9397 (21.7)	1.60 (1.51−1.70)	p<0.001
	N2+	2759 (6.4)	3.56 (3.31−3.84)	p<0.001
UICC stage	Stage I	21786 (50.4)	Reference	
	Stage IIA	11752 (27.2)	1.81 (1.69−1.94)	p<0.001
	Stage IIB	5513 (12.8)	2.45 (2.27−2.65)	p<0.001
	Stage III	4163 (9.6)	4.43 (4.12−4.77)	p<0.001
Tumor grade	G1	8722 (20.2)	Reference	
	G2	28314 (65.5)	1.62 (1.50−1.76)	p<0.001
	G3	5842 (13.5)	2.80 (2.55−3.07)	p<0.001
	Missing	336 (0.8)	1.69 (1.25−2.29)	p=0.001
Menopausal status	Pre−menopausal	10570 (24.5)	Reference	
	Post−menopausal	32260 (74.7)	1.70 (1.58−1.82)	p<0.001
	Unknown − Male	384 (0.9)	3.73 (3.05−4.56)	p<0.001
Residual status	R0	37523 (86.8)	Reference	
	R1 or R2	4877 (11.3)	1.14 (1.05−1.23)	p=0.001
	Missing	814 (1.9)	0.90 (0.74−1.10)	p=0.293
Neoadjuvant ET	No record of neoadjuvant ET	41929 (97)	Reference	
	Record of neoadjuvant ET	1285 (3)	1.22 (1.04−1.42)	p=0.013
Time from diagnosis to first surgery (months)	−	43222 (100)	1.00 (0.96−1.05)	p=0.821
Type of surgery	BCS	32783 (75.9)	Reference	
	Mastectomy	8802 (20.4)	2.69 (2.55−2.85)	p<0.001
	BCS and mastectomy	1629 (3.8)	3.04 (2.75−3.37)	p<0.001

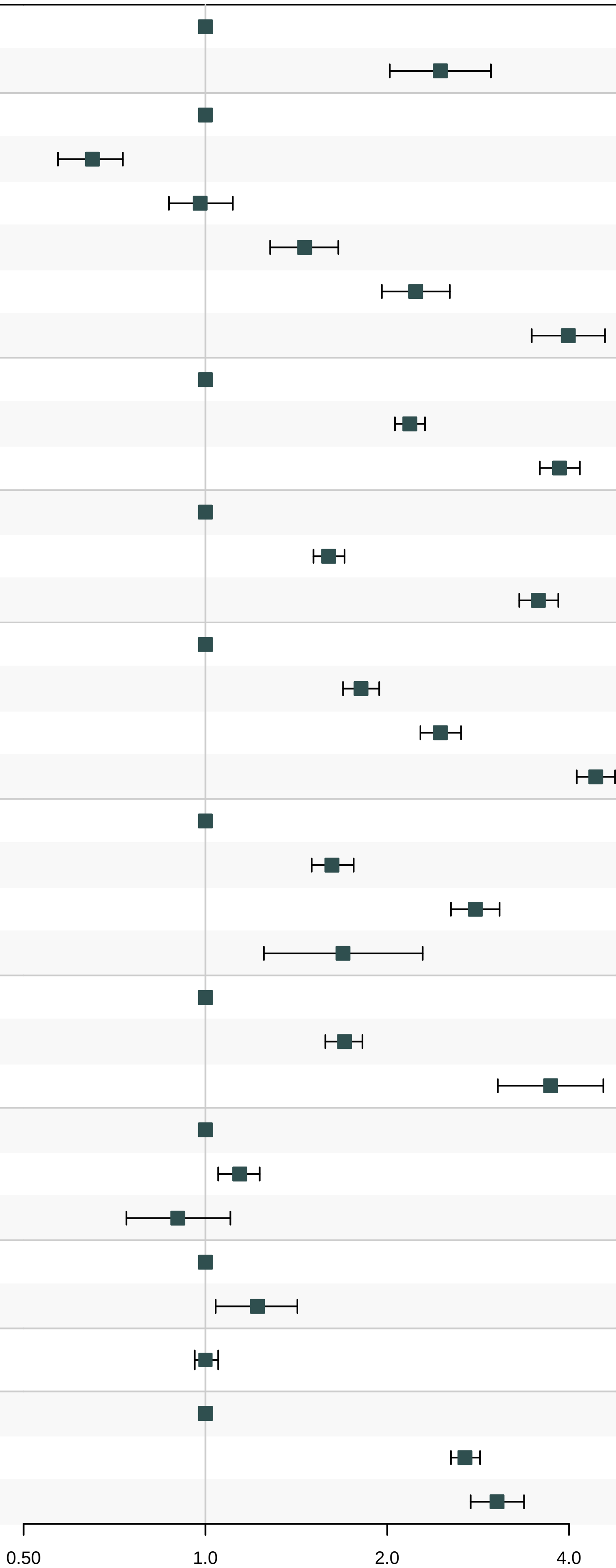

Supplement: Supplementary file 3 [file mmc3.pdf]
