# Supplementary material for: Real-world characteristics and invasive disease-free survival of resected patients with HR-positive/HER2-negative early breast cancer: evidence from German population-based cancer registries
Source: ESMO Real World Data Digit Oncol. 2026 Sep 2;13:100765. doi: 10.1016/j.esmorw.2026.100765 (PMC13571318; doi:10.1016/j.esmorw.2026.100765)
Supplement: Supplementary file 4 [file mmc4.docx]

# Supplement

**eTable S1:** Included federal states of Germany contributing to the analysis, with diagnosis years, number of included cases and mean population size for the respective years

| **Federal State** | **Diagnosis period** | **Number of included cases** | **Population** |
| --- | --- | --- | --- |
| Bavaria^*^ | 2018-2021 | 7059 | 8,131,077 |
| Berlin-Brandenburg | 2017-2021 | 11827 | 6,175,232 |
| Bremen | 2017-2020 | 802 | 681,338 |
| Hessen | 2017-2020 | 3523 | 6,272,576 |
| Hamburg | 2017-2020 | 2588 | 1,842,874 |
| North Rhine-Westphalia | 2017-2021 | 12425 | 17,928,433 |
| Schleswig-Holstein | 2017-2021 | 4473 | 2,904,637 |
| Saarland | 2018-2021 | 525 | 985,934 |
| Total |  | 43,222 | 44,922,101 |

^*^excluding the administrative district of Upper Bavaria and the city and the district of Landshut

**eFigure S1:** Schoenfeld residuals for selected Cox model covariates. Points show observed residuals; lines indicate smoothed trends with 95% confidence intervals.

Abbreviations: ET, endocrine therapy

**eFigure S2:** Overall survival (A) and by menopausal status (B) tumor stage (C) and nodal involvement (D) and with corresponding numbers at risk and survival probabilities (%), including 95% confidence intervals at selected time points

**eFigure S3:** Forest plot of univariable Cox proportional hazards regression models showing the number of patients (N), hazard ratios (HRs) with 95% confidence intervals (CIs), and corresponding p-values

Abbreviations: iDFS, invasive disease-free survival; ET, endocrine therapy; BCS, breast-conserving surgery

**eMethods 1:**

The below rules are used to identify recurrence. If multiple rules are met during a 28-day period, any new recurrence event flags are combined into one single recurrence event, dated at the earliest date, and the type of recurrence is considered as metastatic if any metastatic flags are observed, locoregional if not. This is to avoid double-counting of events that are very likely to be clinically related.

1. New record of CDK4/6 inhibitor or fulvestrant with start date any time after start date of adjuvant therapy

→ **Metastatic recurrence** at start date of CDK4/6 inhibitor or fulvestrant

2. Any new SACT (except CDK4/6 inhibitor or fulvestrant) not meeting the definition of adjuvant therapy^1^

→ **Metastatic recurrence** at start date of new SACT if the new therapy matches the list of treatments list below^2^

→ **Locoregional recurrence** at start date of new SACT if the new therapy does not match the list below^2^

Exceptions to this rule: If a patient receives consecutive regimens meeting the following criteria, the two regimens will be combined into one and the second regimen does not trigger a recurrence

- Both regimens contain at least one of the following: Flurouracil, Epirubicin, Cyclophosphamide, Docetaxel, Paclitaxel, Carboplatin or Trastuzumab
- Neither regimen includes any drug other than those listed in the previous bullet point (except ET)
- There is a gap of less than 60 days between the end of the first regimen and the beginning of the second

3. Record of RT not meeting the definition of adjuvant therapy^1^ starting:
(a) 6+ months after adjuvant ET initiation date; and
(b) 6+ months after latest of either end of adjuvant SACT or end of prior RT

→ **Metastatic recurrence** at start date of RT if treatment is for a non-breast anatomical location

→ **Locoregional recurrence** at start date of RT if treatment is a breast anatomical location

4. Record of new surgery 6+ months after first surgery

→ **Locoregional recurrence** at date of surgery if it is BCS or mastectomy

→ **Metastatic recurrence** at date of surgery if it is surgery related to the liver, lung, brains and/or bones

^1^Adjuvant therapy is defined as starting at the earliest date of any SACT, RT, or ET occurring from the date of surgery and up to 274 days after surgery date

^2^Doxorubicin, Paclitaxel, Capecitabine, Gemcitabine, Vinorelbine, Eribulin, Cyclophosphamide, Docetaxel, Ixabepilone, Cyclophosphamide + Methotrexate + Fluorouracil, Gemcitabine + Carboplatin, Alpelisib + Fulvestrant, Everolimus + Exemestane, Olaparib, Talazoparib, Sacituzumab govitecan (TRODELVY)
